# Is Radiotherapy a Fad?

**Published:** 1905-01

**Authors:** 


### EDITORIAL NOTES AND COMMENTS.

The Business office of THE JOURNAL-RECORD is No. 1007-1008 Century Building.

The Editorial office is 318-319-320 The Prudential.

Address all Business communications to Dr. M. B. Hutchins, Mgr.

Make remittances payable to THE ATLANTA JOURNAL-RECORD OF MEDICINE.

On matters pertaining to the Editorial and Original Communications address Dr. Bernard Wolff, Atlanta.

Reprints of original articles will be furnished at cost price. Requests for the same should always be made *on the manuscript.*

We will present, post-paid, on request, to each contributor of an original article, twenty (20) marked copies of THE JOURNAL-RECORD containing such article.

### IS RADIOTHERAPY A FAD?

BY a fad, we understand the fashion of the day, which to-morrow may be obsolete. How is radiotherapy to be viewed in the light of this definition? Is it a passing fancy held in prominence by spurious testimony as to its merits and doomed to fall into rapid decay when the false witnesses are all discredited and abashed by the light of experience? Or is it a useful, progressive and reliable addition to the weapons given us to combat disease? There are two things necessary to make up a satisfactory answer to this question—more time and more experience. We are of the number who have grown skeptical of professional testimony upon new claimants in the field of materia medica. We have been stuffed with lies, grave, solemn, painstakingly, careful lies for so long that the stomach is fain to puke up its contents when just a whiff of what we are to be fed upon poisons the air.

We have seen so many of these south-sea bubbles break on the surface and leave naught behind save a mephitic odor clinging to those who generated them, that the unbiased testimony of unprejudiced witnesses on clinical observations of drugs and methods is of the nature of light persiflage. Disappointment gives place to disgust and disgust to utter incredulity. Sometimes enthusiasm runs away with sense and reason, sometimes lying case records are deliberately concocted as a professional lure—cipher by whatever means, the result is the same. Are the reports coming in from all Europe and Great Britain and America on the value of the X-rays in the treatment of disease premature, specious and downright lies, or are they, or the most of them true and dependable? The ultra-violet rays and the expensive apparatus required for their production with all their inestimable value in therapeutics do not seem at present to be curing so many cases as formerly. Why is it? Has the supply of violet rays given out, or have all the cases appropriate for treatment by them been cured and the "cause removed," so there are not to be any more?

Is the value of X in the ray equation to be found to be zero or a whole number?

Those who have had experience with radiotherapy and are willing to deliver a plain unvarnished tale are ready to declare that they have found the value of the X, and that it is equal neither to zero nor to infinity. The marvelous reports received from various reliable sources earlier in the course of radiotherapeutic work have been pretty well relegated to the polite fiction shelf while the less gracious truth stands in a compact little volume all to itself. This little volume may be enlarged somewhat and revised from time to time as the years go by, but it is the belief and conviction that it will never get dusty and forgotten but will continue to tell a few facts about a few things in medicine so long as disease holds out to burn. This modest niche which it fills as nothing else has filled heretofore will serve as an answer to the query, is radiotherapy a fad?

